# A high-resolution 3D genome map of kiwifruit provides insights into chromatin architecture and transcriptional activity

**DOI:** 10.1093/hr/uhag076

**Published:** 2026-06-02

**Authors:** Shuangling Xie, Tong Li, Jing Yang, Jingrui Wang, Jinli Gong, Minghui Wang, Xiaoli Hu, Xiaolong Li, Xuepeng Sun

**Affiliations:** State Key Laboratory of Forest Food Resources Development and Utilization, Zhejiang A&F University, Hangzhou 311300, China; Collaborative Innovation Center for Efficient and Green Production of Agriculture in Mountainous Areas of Zhejiang Province, College of Horticulture Science, Zhejiang A&F University, Hangzhou 311300, China; State Key Laboratory of Forest Food Resources Development and Utilization, Zhejiang A&F University, Hangzhou 311300, China; Collaborative Innovation Center for Efficient and Green Production of Agriculture in Mountainous Areas of Zhejiang Province, College of Horticulture Science, Zhejiang A&F University, Hangzhou 311300, China; State Key Laboratory of Forest Food Resources Development and Utilization, Zhejiang A&F University, Hangzhou 311300, China; Collaborative Innovation Center for Efficient and Green Production of Agriculture in Mountainous Areas of Zhejiang Province, College of Horticulture Science, Zhejiang A&F University, Hangzhou 311300, China; State Key Laboratory of Rice Biology, Key Laboratory of Molecular Biology of Crop Pathogens and Insects, Institute of Biotechnology, Zhejiang University, Hangzhou 310058, Zhejiang, China; State Key Laboratory of Forest Food Resources Development and Utilization, Zhejiang A&F University, Hangzhou 311300, China; Collaborative Innovation Center for Efficient and Green Production of Agriculture in Mountainous Areas of Zhejiang Province, College of Horticulture Science, Zhejiang A&F University, Hangzhou 311300, China; State Key Laboratory of Forest Food Resources Development and Utilization, Zhejiang A&F University, Hangzhou 311300, China; Collaborative Innovation Center for Efficient and Green Production of Agriculture in Mountainous Areas of Zhejiang Province, College of Horticulture Science, Zhejiang A&F University, Hangzhou 311300, China; State Key Laboratory of Forest Food Resources Development and Utilization, Zhejiang A&F University, Hangzhou 311300, China; Collaborative Innovation Center for Efficient and Green Production of Agriculture in Mountainous Areas of Zhejiang Province, College of Horticulture Science, Zhejiang A&F University, Hangzhou 311300, China; State Key Laboratory of Forest Food Resources Development and Utilization, Zhejiang A&F University, Hangzhou 311300, China; Collaborative Innovation Center for Efficient and Green Production of Agriculture in Mountainous Areas of Zhejiang Province, College of Horticulture Science, Zhejiang A&F University, Hangzhou 311300, China; State Key Laboratory of Forest Food Resources Development and Utilization, Zhejiang A&F University, Hangzhou 311300, China; Collaborative Innovation Center for Efficient and Green Production of Agriculture in Mountainous Areas of Zhejiang Province, College of Horticulture Science, Zhejiang A&F University, Hangzhou 311300, China

## Abstract

Comprehensive 3D genome maps are essential for understanding transcriptional regulation, yet such resources remain limited for perennial woody crops. Here, we present a high-resolution, tissue-resolved 3D genome atlas of kiwifruit (*Actinidia chinensis*). Using *in situ* Hi-C, we generated chromatin contact maps from leaf and fruit tissues and integrated these data with epigenomic and transcriptomic datasets, including chromatin accessibility, whole-genome DNA methylation, seven histone modifications, and RNA-seq profiles spanning multiple tissues and fruit developmental stages. This integrated dataset enables systematic annotation of genome architecture across multiple spatial scales, including A/B compartments, hierarchical subcompartments, TAD-like domains, and chromatin loops. Global features of 3D genome organization are broadly similar between tissues, while quantitative variation is observed at finer scales, such as subcompartment rank, domain insulation strength, and loop detection frequency. Integration with genomic and epigenomic features reveals consistent associations between chromatin states and spatial organization, providing a reference framework for interpreting plant genome architecture in a perennial context. We further map tissue-specific gene sets onto the 3D genome landscape and describe their spatial distributions relative to compartments, domains, and loop anchors, offering a view of how transcriptional programs relate to higher-order chromatin organization. Together, this work establishes an integrative, high-resolution 3D genome resources for a woody perennial fruit crop, and supports future functional, evolutionary, and applied research in kiwifruit and other perennial species.

## Introduction

The 3D genome architecture plays a central role in orchestrating transcriptional programs and epigenetic landscapes in eukaryotes [1, 2]. It operates at multiple spatial scales, from megabases to kilobases, and coordinates chromatin contacts to ensure cell type-specific gene control [3, 4]. Advances in chromosome conformation capture technologies, particularly Hi-C, have revolutionized our understanding of this hierarchical organization [5, 6], revealing three principal tiers of genome folding: (i) megabase-scale A/B compartments, which partition the genome into transcriptionally active (A) and repressive (B) nuclear territories, each marked by distinct histone modification profiles [7]; (ii) topologically associating domains (TADs), which define self-interacting chromatin domains (0.1–1 Mb) that constrain enhancer–promoter communication [7, 8]; and (iii) chromatin loops, which establish precise, often cell state-specific contacts between distal regulatory elements and target gene promoters [1, 3, 9–12].

The hierarchical organization of the 3D genome—including A/B compartments, TAD-like domains, and chromatin loops—appears to be evolutionarily conserved across plant species [13–15]. A/B compartments are strongly associated with transcriptional activity, chromatin accessibility, and histone modifications, and their dynamic switching across developmental stages contributes to transcriptional plasticity [16]. Intriguingly, plant genomes have evolved TAD-like chromatin domains despite lacking metazoan architectural proteins such as CTCF. These domains often coincide with transitions in epigenetic states and demarcate gene regulatory units [17, 18]. Typically classified as active, repressive, or intermediate, the functional dynamics of these domains—such as their stability across the cell cycle, tissue-specific organization, and direct regulatory impacts on gene expression—remain incompletely understood [19, 20]. Chromatin looping, though less well characterized in plants than in animals, has been increasingly implicated in long-range transcriptional regulation [21, 22]. However, its mechanistic roles and functional relevance in coordinating plant-specific developmental programs remain largely unresolved [23].

While foundational frameworks of plant 3D genome organization have emerged from model systems and annual crops, perennial species, particularly long-lived fruit trees, remain understudied [24–28]. The dioecious perennial kiwifruit (*Actinidia chinensis*), a woody species of significant agronomic and nutritional importance, serves as a good model for investigating chromatin architecture in species with extended life cycles and complex developmental programs [29, 30]. Seminal Hi-C analyses in kiwifruit revealed conserved A/B compartmentalization, a remarkably high density of TAD-like domains, and tissue-specific chromosomal reorganization. Notably, many TAD-like boundaries encompass tandem-duplicate gene clusters (TDGCs), suggesting a mechanistic link between 3D chromatin architecture and genome evolution [31]. Comparative studies further demonstrated that kiwifruit lacks the large gene-to-gene loop (GGL) arrays typical of large genomes like wheat, yet harbors a distinct class of TDGC-associated TAD-like domains. These are independent of H3K27me3, conserved across *Actinidia* species, enriched for young and highly coexpressed duplicates, and associated with rapid tandem gene birth [31–33]. Together, these findings position kiwifruit as a unique system where higher order genome folding associates with both transcriptional regulation and gene family expansion. However, previous studies have primarily focused on structural features and evolutionary patterns, leaving unresolved the tissue-specific regulation of 3D genome organization and its integration with dynamic epigenomic states, chromatin accessibility, and gene expression [34].

Here, we generated high-resolution *in situ* Hi-C maps from kiwifruit leaf and fruit tissues and integrated them with epigenomic datasets, including chromatin accessibility (ATAC-seq), whole-genome DNA methylation, seven histone modifications (H3K4me3, H3K27me3, H3K36me3, H3K9ac, H3K27ac, H3K9me3, and H3K4me1), and transcriptomic profiles across multiple tissues and fruit developmental stages. This comprehensive dataset enables multiscale analyses of genome architecture—including A/B compartments, subcompartments, TAD-like domains, and chromatin loops—across tissues, and providing an integrated view of chromatin organization, epigenomic regulation, and spatiotemporal gene expression in a perennial fruit crop.

## Results

### Three-dimensional genome organization in kiwifruit leaf and fruit tissues

**Figure 1:**
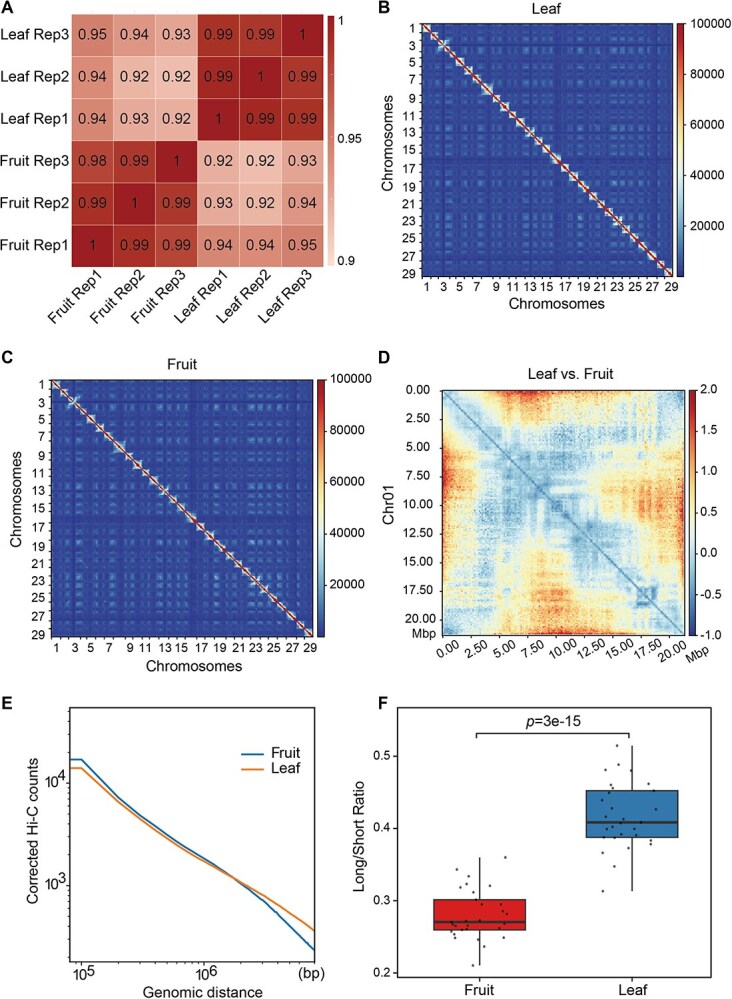
Tissue-specific chromatin contact landscapes in kiwifruit. **(A)** Reproducibility of Hi-C data. A heatmap shows the pairwise Pearson correlation coefficients (PCCs) for all Hi-C replicates, demonstrating high reproducibility within leaf and fruit datasets (PCC > 0.9). **(B and C)** Genome-wide chromatin contact matrices for leaf and fruit tissues, visualized at a 100-kb resolution. These matrices reveal distinct spatial organization without evidence of a Rabl chromatin configuration. **(D)** Chromosome 1 difference map at 25-kb resolution. The map highlights regions with higher contact frequency in fruit (blue) and leaf (red), revealing tissue-specific variations in chromatin structure. **(E)** Contact decay curves. Corrected Hi-C contact counts are plotted against genomic distance, showing that fruit chromatin has more short-range interactions, while leaf chromatin exhibits increased long-range interactions. **(F)** Boxplot of long/short-range contact ratios. This plot shows a statistically significant enrichment of long-range contacts in leaf samples compared to fruit (Wilcoxon test, *P* = 3e–15).

To characterize the 3D chromatin architecture of kiwifruit, we performed high-resolution *in situ* Hi-C sequencing on leaf and fruit tissues, with three biological replicates per tissue. Sequencing generated ~6.51 billion paired-end reads (2 × 150 bp) across all samples, with each library yielding 826–1203 million reads, corresponding to sequencing depths of ~155–248× (Supplementary Table S1). Contact matrices were generated using the HiCExplorer pipeline and normalized to a 2-kb resolution using an iterative correction approach (Supplementary Table S2). Biological replicates exhibited high reproducibility, with stratum-adjusted correlation coefficients (SCCs) exceeding 0.92 (Fig. 1A), and Pearson correlation coefficients >0.98 (Supplementary Fig. S1A).

Hi-C contact matrices displayed strong signal intensity along the diagonal across all chromosomes in both leaf and fruit tissues, consistent with the expected distance-dependent decay of chromatin interactions (Fig. 1B and C). No evidence of Rabl chromosome configuration was detected in either tissue, in agreement with previous reports that such configurations are often absent in species or cell types with small, numerous chromosomes that can freely reorient during interphase [35]. Comparison of normalized contact matrices revealed differences in interaction patterns between the two tissues, indicating regional variability in chromatin connectivity (Fig. 1D). Analysis of interaction frequency decay suggested distinct distributions of short- and long-range contacts, with fruit tissue showing relatively higher proportions of short-range interactions (<100 kb) and leaf tissue exhibiting comparatively more long-range interactions (>1 Mb) (Fig. 1E; Supplementary Fig. S1B). Consistently, the ratio of long- to short-range interactions was higher in leaf tissue (*P* = 3e–15, Fig. 1F; Supplementary Fig. S1C). Taken together, these results indicate that overall principles of chromatin folding are broadly similar between leaf and fruit tissues, while quantitative differences in interaction distance distributions are detectable. Given that this study is limited to two tissue types, these observations provide an initial view of chromatin organization in kiwifruit and may not fully capture the diversity of 3D genome configurations across other tissues or developmental contexts.

### Genomic and epigenomic profiles of chromatin subcompartments

**Figure 2:**
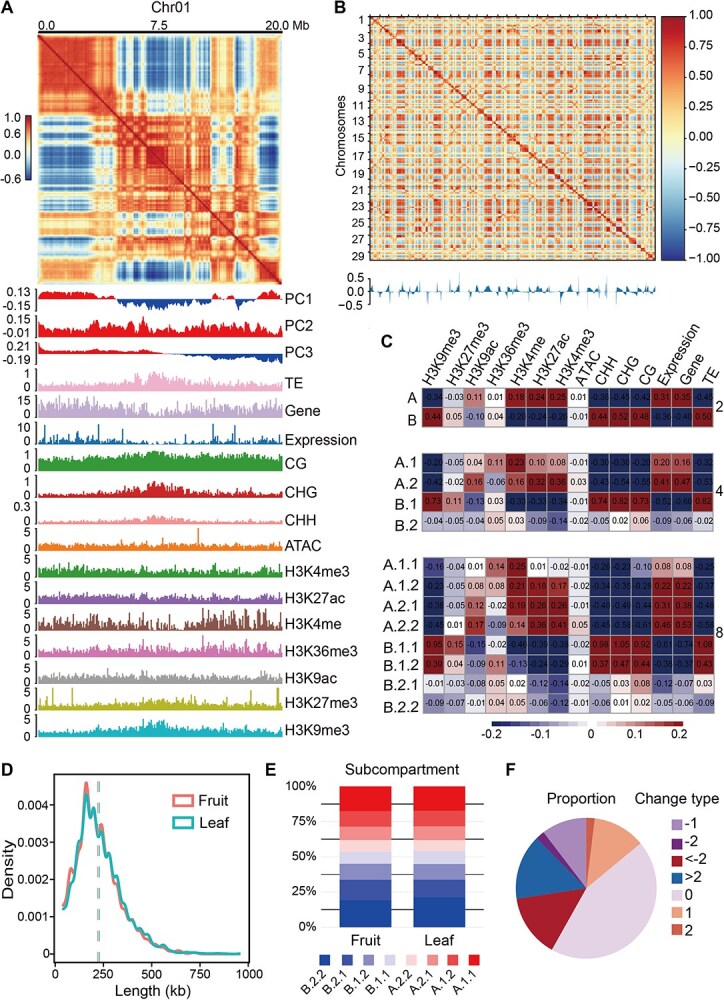
Subcompartment architecture and associated genomic features in the kiwifruit genome. **(A)** Pearson correlation matrix for Hi-C contacts on chromosome 1, at 100-kb resolution, with the PC1-based A/B compartment signal aligned with gene density, epigenetic marks, and transcription, illustrating the correlation of compartment status with genomic features. **(B)** A genome-wide visualization of A/B compartments in leaf tissue at 250-kb resolution, where positive PC1 values denote the active A compartment and negative values denote the inactive B compartment. **(C)** Enrichment profiles of various genomic and epigenomic features (gene expression, TEs, DNA methylation, histone modifications, and chromatin accessibility) across subcompartments, revealing their distinct functional characteristics. **(D)** Size distribution of subcompartments, showing an average length of ~225 kb. **(E)** Proportional representation of each subcompartment type (A1.1–B2.2) in fruit and leaf tissues. B-type subcompartments, which are associated with repressive marks, constitute a significant portion (55%–60%) of the genome. **(F)** Analysis of subcompartment dynamics between fruit and leaf tissues, showing the proportion of genomic bins exhibiting different degrees of hierarchical change. Bars indicate bins with unchanged subcompartment assignments (‘0’), shifts spanning one (‘±1’) or two (‘±2’) hierarchical levels, or transitions involving more than two levels (‘> ±2’). Positive (‘+’) and negative (‘−’) values denote shifts toward higher (e.g. A.1.1) or lower (e.g. B.2.2) subcompartment ranks, respectively.

To explore the hierarchical chromatin organization and its relationship with genomic and epigenomic features in kiwifruit, we performed systematic subcompartment analysis across multiple resolutions. Using chromosome 1 as an example, we first generated a Pearson correlation matrix of Hi-C contacts at 100-kb resolution and conducted principal component analysis (PCA) to identify broad A/B compartments (Fig. 2A). For each chromosome, the principal component (PC1, PC2, or PC3) that best captured the compartmental checkerboard pattern was selected as the compartment axis. This PC-based classification was then extended genome-wide, and the resulting A/B compartment landscapes in leaf and fruit tissues were visualized at 250-kb resolution (Fig. 2B; Supplementary Fig. S2A). The PCA signal revealed alternating A/B compartments with relatively diffuse boundaries, consistent with gradual compartment transitions commonly observed in plant genomes [36].

PC values were then used to assign A and B compartments, which were evaluated in relation to transposable element (TE) distribution, gene density, DNA methylation, histone modifications, and transcriptional activity. To resolve finer scale chromatin organization beyond broad A/B domains, we applied Calder [37] to Hi-C matrices at both 100- and 40-kb resolutions to infer nested subcompartments. At 40-kb resolution, Calder identified eight subcompartment classes (from highest rank A1.1 to lowest rank B2.2) with an average length of ~225 kb (Fig. 2C and D), which showed strong concordance with chromatin states. When aggregated to 100-kb resolution, subcompartments expanded to an average of ~595 kb (Supplementary Fig. S2B). Correlation analyses revealed that A-type subcompartments were generally associated with active histone marks (e.g. H3K4me, H3K4me3, H3K27ac) and higher transcriptional activity, whereas B-type subcompartments were enriched for TEs, higher levels of CG, CHG, and CHH methylation, and repressive histone marks (e.g. H3K9me3, H3K27me3). These structure–epigenome associations were notably stronger at the finer 40-kb resolution (Supplementary Fig. S2C). Moreover, ATAC-seq signals showed limited differentiation between A and B compartments at both resolutions, which may reflect the sparse and localized nature of chromatin accessibility peaks and the tendency for compartment-level differences to be dampened by bin-level averaging at 40–100 kb.

Comparative analyses of subcompartment assignments between leaf and fruit tissues revealed a high degree of overall concordance. At 100-kb resolution, more than 60% of genomic bins showed identical subcompartment assignments between the two tissues (Supplementary Fig. S2D), suggesting substantial conservation of higher order chromatin organization. Increasing the resolution to 40 kb did not significantly alter this pattern, indicating general stability of subcompartment architecture across the examined tissues. Genome-wide assessments further showed broadly similar subcompartment compositions in leaf and fruit, with B-type subcompartments accounting for ~55%–60% of the genome in both cases (Fig. 2E), consistent with the prevalence of repressive chromatin domains in plant genomes.

To further quantify variability in subcompartment assignments, we categorized genomic regions into three classes based on their degree of change across tissues: stable regions (44.14%) retained identical assignments; modulated regions (26.40%) exhibited limited shifts involving one or two subclass changes; and rewired regions (29.46%) showed more extensive transitions across multiple subcompartment levels (Fig. 2F). Together, these analyses indicate that while much of the subcompartment architecture is conserved between leaf and fruit tissues, a subset of regions displays quantitative variability. Integrating multiresolution subcompartment mapping (40–250 kb) with epigenomic profiles thus provides a framework for describing chromatin organization in kiwifruit and establishes a reference for future studies examining spatial genome regulation across additional tissues or developmental contexts.

### Hierarchical organization and epigenomic signatures of TAD-like domains

**Figure 3:**
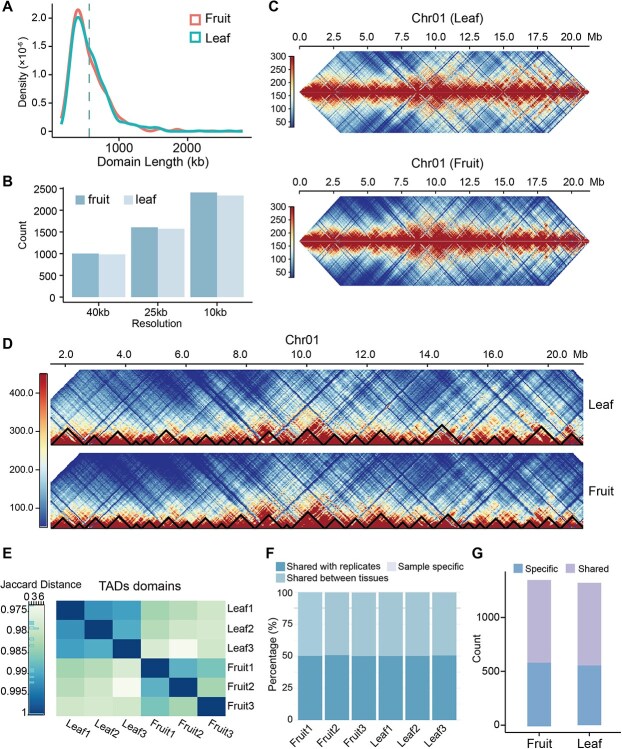
Identification and conservation of TAD-like domains in kiwifruit leaf and fruit tissues. **(A)** Length distribution of TAD-like domains identified at 40-kb resolution in leaf tissue. **(B)** Total number of TAD-like domains identified at 40-, 25-, and 10-kb resolution in leaf tissue. **(C**) Hi-C contact maps of chromosome 1 at 40-kb resolution showing TAD-like domains annotated in leaf and fruit tissues. **(D)** Genome browser view of selected regions showing conserved and tissue-specific domain boundaries between tissues. **(E)** Jaccard distance matrix illustrating boundary similarity and reproducibility across samples. **(F)** Proportion of shared and unique TAD-like domains across biological replicates and tissues. **(G)** Histogram showing pairwise domain overlap percentages between leaf and fruit samples.

We systematically characterized TAD-like structures in leaf and fruit tissues across multiple resolutions. Using HiCExplorer, chromatin domains were identified at 40-, 25-, and 10-kb resolutions. In leaf tissue, this multiscale analysis detected 982, 1573, and 2338 TAD-like domains, with average sizes of 565 ± 42, 365 ± 28, and 250 ± 19 kb, respectively (Fig. 3A and B; Supplementary Fig. S3A). Smaller domains were frequently nested within larger domains, indicating a hierarchical organization of chromatin folding. Similar multiscale domain structures have been reported in other eukaryotic systems, suggesting that hierarchical chromatin organization represents a broadly conserved feature of genome architecture.

Comparisons between leaf and fruit tissues at 40- and 25-kb resolutions revealed generally similar TAD-like patterns, with only minor differences in boundary positions and domain sizes (Fig. 3C; Supplementary Fig. S3B). Overall, domain organization appeared largely conserved between the two tissues (Fig. 3D). Jaccard similarity analysis showed high reproducibility between biological replicates (mean Jaccard index = 0.99) and substantial concordance between tissues (mean Jaccard index = 0.97) (Fig. 3E; Supplementary Fig. S3C–F). Pairwise comparisons indicated that ~49% of domains were shared between leaf and fruit tissues, ~50% were conserved within biological replicates of the same tissue, and ~1% were detected predominantly in one tissue (Fig. 3F and G). These results indicate that the overall TAD-like domains are stable across the examined tissues, while a limited subset of domains shows variability. Broader sampling across additional tissues, developmental stages, or environmental conditions will be required to more fully assess context-dependent chromatin dynamics.

**Figure 4:**
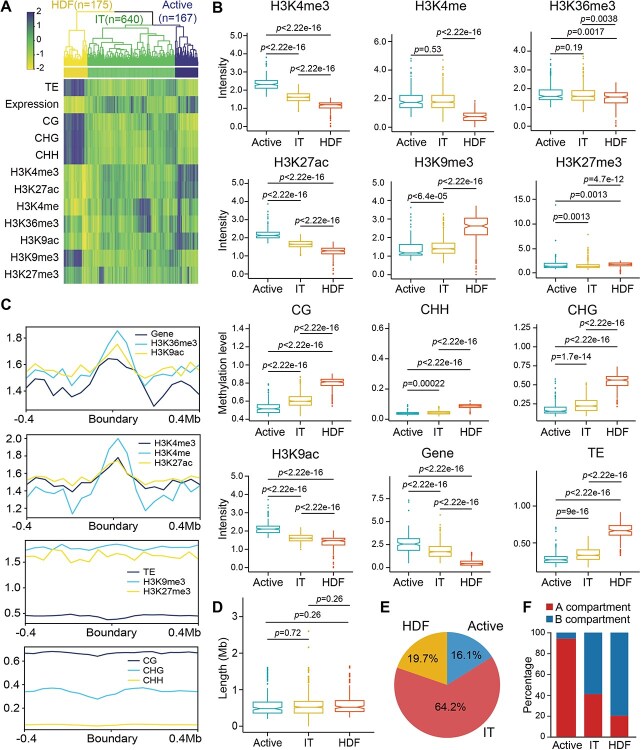
Classification and epigenomic profiling of TAD-like domains in leaf tissue at 40-kb resolution. **(A)** Hierarchical clustering divides domains into Active, IT, and HDF types based on multi-omics features. **(B)** Comparison of genomic and epigenomic features across the three domain types. HDF domains are typically enriched for TEs and repressive histone mark H3K9me3, corresponding to compact chromatin regions with limited long-range interactions (see also panel A). **(C)** Aggregate signal plots showing the distribution of active and repressive histone modifications around domain boundaries. **(D)** Domain length comparison across Active, IT, and HDF classes. **(E)** Proportion of each domain type in the genome. **(F)** Relationship between domain classes and A/B compartments.

To examine the relationship between chromatin domain organization and epigenomic features, we integrated leaf Hi-C data at 40- and 25-kb resolutions with genomic and epigenomic datasets, including gene density, TE content, DNA methylation, and histone modifications. Following a classification previously established in pepper [25], TAD-like domains were grouped into three categories: (i) active domains (*n* = 167), enriched for highly expressed genes and active histone marks such as H3K4me3, H3K4me1, H3K36me3, and H3K9ac; (ii) heterochromatin-driven folding (HDF) domains (*n* = 175), associated with high TE content, elevated levels of DNA methylation (CG, CHG, CHH), and repressive histone marks including H3K9me3 and H3K27me3; and (iii) intermediate type (IT) domains (*n* = 640), characterized by low gene expression and a mixture of both active and repressive epigenetic signatures (Fig. 4A and B). Domain classification at 40-kb resolution provided clearer separation among these categories than at 25-kb resolution (Supplementary Fig. S4A and B), highlighting the utility of higher resolution Hi-C data for resolving chromatin states. Genome-wide distribution further showed that HDF domains frequently coincide with TE-rich and H3K9me3-enriched regions, consistent with their predominant association with B compartments (Fig. 4B and F).

We next examined genomic and epigenomic features at TAD-like domain boundaries. Consistent with observations in *Drosophila* and pepper [25, 38, 39], boundaries were significantly enriched for genes and active chromatin marks, including H3K4me3, H3K27ac, H3K4me1, H3K36me3, and H3K9ac. In contrast, repressive features such as TEs, DNA methylation, H3K9me3, and H3K27me3 did not show significant enrichment at boundaries relative to random genomic regions (Fig. 4C; Supplementary Fig. S4E). Genome-wide quantification revealed that IT domains comprised the majority of the genome (~64.2%), followed by HDF domain (~19.7%) and Active domain, although the lengths of different domain types were indistinguishable (Fig. 4D and E). Compartment analysis further indicated a strong correspondence between domain types and nuclear organization, with HDF domains preferentially located in B compartment and Active domains enriched in A compartment (Fig. 4F).

### Gene expression variation and chromatin 3D reorganization

**Figure 5:**
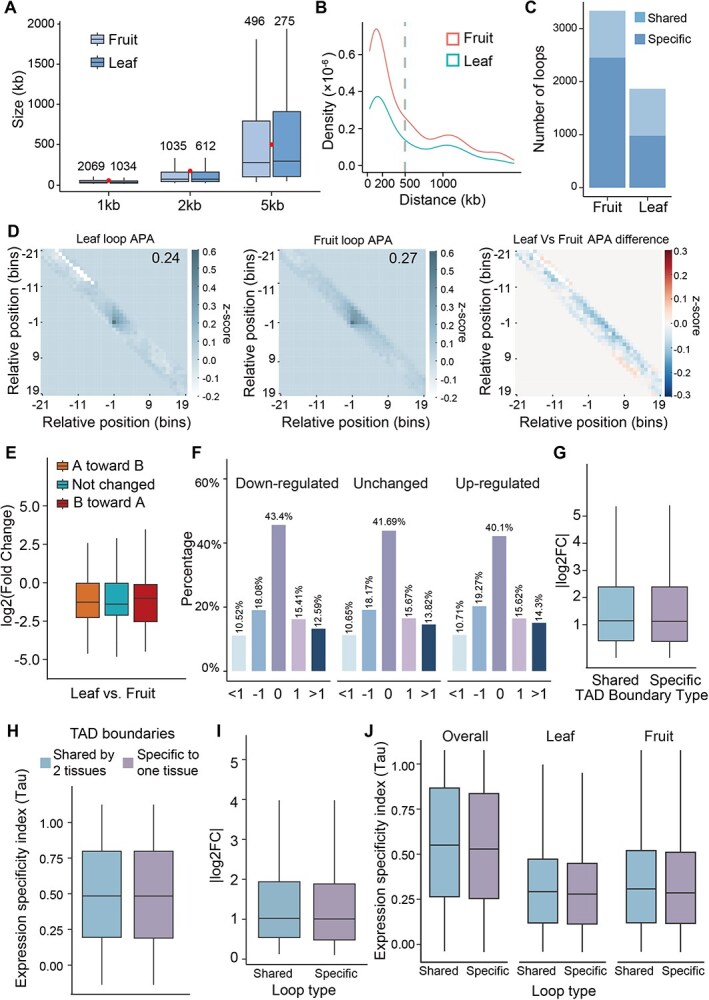
Relationship between chromatin architecture and transcriptional variation in kiwifruit. **(A)** Comparison of average loop sizes across resolutions, showing that loop span increases as resolution decreases. **(B)** Size distribution of chromatin loops identified at 1-, 2-, and 5-kb resolutions. **(C)** Number of chromatin loops detected in leaf and fruit tissues; approximately half of the leaf loops are also observed in fruit. **(D)** APA of loop anchors in fruit and leaf tissues, indicating strong signal enrichment and reproducibility. **(E)** Relationship between subcompartment rank changes (≥2 ranks) and gene expression level. **(F)** Cataloging the number of differentially expressed genes associated with subcompartment transitions. Definition for subcompartment transition is described in Fig. 2F. **(G)** Comparison of the number of DEGs near tissue-specific vs conserved TAD boundaries; no statistical significance was observed. **(H)** Tau expression specificity index for genes located near tissue-specific and conserved TAD boundaries, showing no significant difference. **(I)** Comparison of gene expression in chromatin loops categorized as shared or tissue-specific, revealing no significant difference. **(J)** Tau index comparison for genes associated with shared and tissue-specific loop anchors, indicating overall transcriptional stability.

To examine chromatin looping in kiwifruit, we analyzed Hi-C data from leaf and fruit tissues using the Mustache algorithm [40] applied to aggregated contact matrices (1, 2, and 5 kb) from three biological replicates per tissue. This analysis identified 1660 nonredundant loops in leaf tissue and 3117 nonredundant loops in fruit tissue (Fig. 5A). Approximately 50% of leaf loops overlapping with those in fruit (Fig. 5B and C). Notably, kiwifruit chromatin loops were generally short in genomic span, with >80% covering <500 kb, differing from loop size distributions reported in pepper and human genomes [25, 41]. The effective detection of chromatin loops was resolution-dependent, with the average loop span increasing from ~48 kb at 1-kb resolution to ~500 kb at 5-kb resolution (Fig. 5B; Supplementary Fig. S5A). To assess the robustness of loop detection, we independently validated Mustache-identified loops using hicDetectLoops (HiCExplorer) across multiple resolutions (5, 10, 25, and 40 kb) (Supplementary Table S3). Aggregate peak analysis (APA) revealed clear enrichment of interaction signals at loop anchors in both tissues (Fig. 5D; Supplementary Fig. S5B), supporting the reliability of loop calls. On average, loops detected in fruit exhibited slightly higher interaction intensities than those in leaf tissue, though this difference was not statistically significant (*t*-test, *P* = 0.73). These results suggest that the higher number of loops detected in fruit may primarily reflect increased local contact density rather than systematic differences in anchor strength. To further characterize loop features, we annotated loop anchors with major genomic and epigenomic elements. Among the 521 loop anchors identified in leaf tissue, 38.96% overlapped promoter regions (transcription start site (TSS) ± 2 kb), 51.44% overlapped gene bodies, and 96.16% were located within TE-rich regions. Only a subset of anchors colocalized with active regulatory features, including ATAC-seq peaks (8.06%) and histone marks such as H3K27ac (43.95%) and H3K4me3 (36.85%) (Supplementary Fig. S5C).

We next examined potential associations between chromatin organization and transcriptional variation. Differentially expressed genes (DEGs) were neither enriched nor depleted in regions undergoing A/B compartment switching (Fisher’s exact test, *P* > 0.1), and their expression levels were not significantly affected by subcompartment transitions (Wilcoxon rank-sum test, *P* > 0.05; Fig. 5E). In contrast, genomic regions transitioning toward higher ranked subcompartments tended to show increased expression, whereas shifts toward lower ranked subcompartments were associated with reduced expression (Fig. 5F). Consistently, stratifying genes based on expression changes revealed broadly similar compartmental distributions among upregulated, downregulated, and transcriptionally stable genes (Fig. 5F). With respect to TAD-like domains and their boundaries, no significant enrichment of DEGs was observed at tissue-variable boundaries (Fisher’s exact test, *P* = 0.71), and DEG expression levels were comparable between boundary categories (*t*-test, *P* = 0.24) (Fig. 5G). Likewise, expression profiles were largely similar between conserved and tissue-variable domains, as reflected by comparable tissue specificity index (Tau) values (Fig. 5H).

We further assessed associations between chromatin loop organization and transcriptional variation by classifying loops as shared (*n* = 1480) or detected predominantly in one tissue (*n* = 3907). Both shared (42.8%) and tissue-variable (41.7%) loops exhibited similar overlap with DEGs relative to the genomic background, with no significant enrichment in either category (Fisher’s exact test, *P* = 0.477). Although shared loops showed a slightly higher proportion of DEGs compared with tissue-variable loops (Fisher’s exact test, *P* = 0.02), Tau-based analyses indicated comparable expression patterns between loop categories (Fig. 5I and J). Taken together, these results indicate that while aspects of genome organization are associated with transcriptional activity, variability in chromatin structure does not appear to correspond to widespread changes in gene expression between the leaf and fruit tissues examined here in kiwifruit.

### Chromatin organization at fruit-specific gene loci

**Figure 6:**
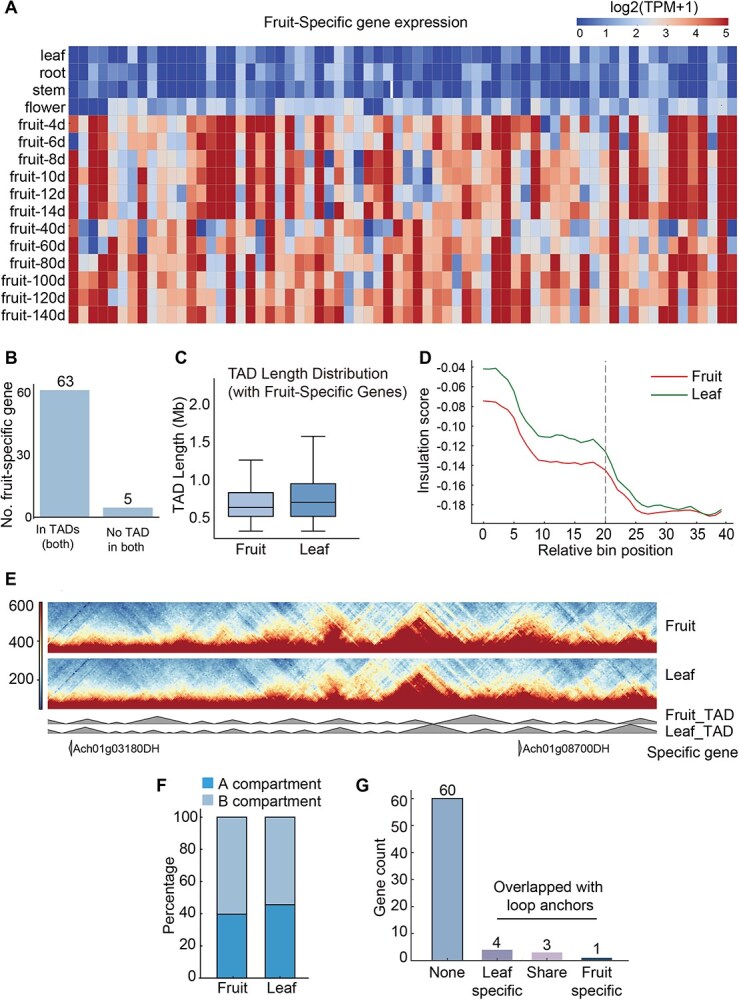
Chromatin organization of fruit-specific genes in kiwifruit. **(A)** Heatmap showing the expression profiles (log₂[TPM + 1]) of 68 fruit-specific genes across 14 tissues and developmental stages of kiwifruit. Genes were consistently expressed in fruit samples (12 stages) and showed minimal expression in root, leaf, stem, and flower tissues. **(B)** Histogram indicating the number of fruit-specific genes located within TAD-like domains identified in both fruit and leaf (*n* = 63) vs those not overlapping any annotated TAD (*n* = 5). **(C)** Boxplot comparing the lengths of TADs harboring fruit-specific genes in fruit and leaf tissues. No significant difference was observed between the two tissues (Wilcoxon test). **(D)** Line plot showing the average insulation score surrounding TAD boundaries associated with fruit-specific genes in fruit and leaf. The *x*-axis denotes the bin distance from the boundary center (bin 20). Lower insulation scores in fruit indicate stronger boundary insulation. **(E)** Selected Hi-C contact maps for a genomic locus containing fruit-specific genes in fruit (top) and leaf (bottom) tissues. TAD boundaries are annotated below each matrix, illustrating tissue-specific domain reorganization. **(F)** Stacked bar plots showing the distribution of fruit-specific genes across A and B compartments in fruit and leaf tissues. Over 60% of genes are located in the B compartment in fruit, while their distribution in leaf is approximately balanced. **(G)** Histogram showing the distribution of fruit-specific genes with respect to chromatin loops. The majority of genes (60 of 68; 88.2%) do not overlap with any detected loops, whereas a small subset colocalizes with loops that are leaf-specific (4 genes; 5.9%), shared between tissues (3 genes; 4.4%), or fruit-specific (1 gene; 1.5%).

Using RNA-seq data from diverse tissues and developmental stages of kiwifruit ‘Hongyang’—including root, leaf, stem, flower, and 12 fruit developmental time points—we applied stringent criteria to identify genes with predominant expression in fruit tissues. Genes were retained if they showed consistently high expression across all fruit stages (TPM >1), low or undetectable expression in nonfruit tissues (TPM <0.5), a tissue-specificity index (Tau) exceeding 0.6, and fruit-derived transcripts accounting for >80% of total expression. This yielded 68 high-confident fruit specifically expressed genes (Fig. 6A). We mapped these genes onto Hi-C–derived chromatin architectures of both fruit and leaf tissues. The majority (92.6%, 63/68) were located within TAD-like domains in both tissues (Fig. 6B), with no significant difference in domain length between fruit (638.6 ± 333.2 kb) and leaf (686.3 ± 308.0 kb; *P* = 0.43) (Fig. 6C). To assess boundary strength, we calculated average insulation scores around TAD borders harboring fruit-specific genes. Boundaries in fruit exhibited lower insulation scores than in leaf (Fig. 6D). The Hi-C contact maps further illustrated differences in local folding patterns between tissues at selected regions (Fig. 6E). At the compartment level, >60% of fruit-specific genes were assigned to the B compartment in fruit, whereas their distribution in leaf was nearly balanced between compartments A and B (Fig. 6F). Analysis of long-range chromatin interactions further showed that 12% of fruit-specific genes overlapped with loop anchors—four associated with loops detected predominantly in leaf, three with shared loops, and one with a loop detected predominantly in fruit (Fig. 6G). These results suggest that activation of some fruit-specific genes might be partly associated with changes in local chromatin organization.

## Discussion

We generated high-resolution Hi-C maps of kiwifruit in leaf and fruit tissues and integrated them with multi-omics data to characterize 3D genome organization and its relationship to gene expression. Across tissues, the global features of chromatin architecture—including A/B compartments, subcompartments, TAD-like domains, and chromatin loops—were broadly similar. At finer scales, quantitative variation was observed, including subcompartment rank changes in a subset of the genome, differences in domain insulation strength, and variation in detected loop numbers. Fruit-specific genes were frequently located within B compartments and insulated domains and showed limited overlap with chromatin loop anchors, indicating that local chromatin context, particularly at the domain scale, is a prominent feature at these loci.

Hi-C analyses revealed clear A/B compartmentalization, with subcompartment identities closely aligned to genomic and epigenomic features: A-type regions were generally gene-rich and associated with active histone marks, whereas B-type regions were enriched for TEs, DNA methylation, and repressive chromatin marks. Over 60% of subcompartments were conserved between leaf and fruit tissues, while ~30% exhibited modest rank shifts. These shifts showed graded associations with gene expression levels, although additional analyses indicated that transcriptional variation alone does not explain most subcompartment dynamics, consistent with a largely correlative relationship between spatial organization and transcriptional output. TAD-like domains displayed hierarchical nesting and were largely conserved across two tissues, with boundaries enriched for active chromatin features. Classification of domains identified three categories—Active, HDF, and IT—with IT domains comprising ~64% of the genome. Although chromatin loops were more frequently detected in fruit, their association with differential gene expression or tissue specificity was limited. Overall, these observations suggest that domain-scale organization represents a stable structural framework, within which transcriptional variation often occurs.

These findings raise broader questions regarding the extent to which the chromatin features observed in kiwifruit are shared with other plant species, particularly large and polyploid crops, and what molecular mechanisms underlie domain-level insulation in plant genomes. Our results are consistent with an emerging view that plant gene regulation relies heavily on chromatin context rather than pervasive loop-mediated enhancer–promoter interactions. In addition, because ATAC-seq signals were analyzed at 40–100 kb to match Hi-C resolution, fine-scale promoter or enhancer accessibility could not be resolved; higher resolution assays will be needed to address these questions.

Similar patterns—where large-scale folding is stable while transcription remains flexible—have been reported in tomato, rice, and pepper [25, 42–44]. In hexaploid wheat, e.g. a large number (~18 600) of TAD-like domains were identified, yet only a small fraction (<6%) of RNAPII-associated loops overlapped differentially expressed genes [32]. Likewise, drought-responsive Hi-C analyses in rice showed limited correspondence between stress-induced loops and transcriptional changes [45], comparable to the low overlap observed between loops and fruit–leaf expression differences in kiwifruit.

While most 3D genome studies have focused on annual herbaceous species, comparable analyses in woody or perennial plants remain limited. Available Hi-C datasets for grape and poplar have primarily supported genome scaffolding or coarse architectural characterization [46, 47]. The kiwifruit datasets presented here therefore provide one of the few integrative 3D genome resources for a perennial woody fruit crop and establish a reference for future comparative and functional studies. Observations from grasses such as rice, *Brachypodium*, and cotton indicate that chromatin loops are associated with only a subset of transcriptional changes, whereas the majority of transcriptional variation is more closely linked to domain-level organization and local chromatin architecture across plant lineages [45, 48–52].

Recent biophysical studies have shown that heterochromatin protein ADCP1 can oligomerize on H3K9me3-marked nucleosomes to form liquid-like condensates tethered to the nuclear periphery by CRWN1/4 lamina proteins [53, 54]. This mechanism stabilizes repressive chromatin in *Arabidopsis* and may provide a conceptual framework for understanding the insulated B-type and IT domains prevalent in the kiwifruit genome [18, 54]. Consistent with this model, single-molecule imaging indicates that plant cohesin is capable of loop extrusion but operates under constraints imposed by PDS5/WAPL-mediated residence-time control [26, 55–58]. In *Arabidopsis*, *pds5* multigene mutants show a surge in TAD-like numbers with minimal transcriptional change [26]. A comparable pattern in polyploid wheat suggests that absolute loop output becomes increasingly dispensable as gene copy number rises and insulation strength intensifies [32]. In maize, pepper, and wheat, high-density TE islands act as nucleation points for nested IT domains [12, 23, 25, 32, 44]. In kiwifruit, TE- and H3K9me3-enriched regions displayed compact 3D configurations with reduced long-range interactions, resembling heterochromatic domains reported in other plant genomes. APA analysis centered on relaxed pericentromeric regions showed smooth distance-dependent decay patterns without focal interaction enrichment in both leaf and fruit tissues (Supplementary Fig. S6), indicating that these domains maintain compact yet structurally stable conformations across tissues. These results support the view that TE-rich heterochromatin contributes to local chromatin compaction and the maintenance of repressive B-type compartments, rather than acting as focal hubs of looping interactions. Consistent with this interpretation, TAD boundaries themselves were not enriched for TEs or repressive histone marks, suggesting that heterochromatin contributes to insulation indirectly through compact domain interiors rather than by directly specifying boundary locations.

In summary, we present an integrative, tissue-based 3D genome atlas for the perennial fruit crop kiwifruit, combining high-resolution Hi-C with epigenomic and transcriptomic datasets. Multiscale analyses indicate that fruit-specific transcription is commonly associated with insulated chromatin domains within B-type compartments, whereas long-range looping shows limited correspondence with expression differences. These conclusions are based on bulk Hi-C and population-averaged epigenomic data and therefore do not capture cell type-specific variability. Moreover, insulation strength depends on algorithmic definitions, and functional relationships between chromatin organization and transcription remain to be experimentally tested. Expanding these analyses to additional tissues, developmental stages, and environmental conditions, as well as applying single-cell Hi-C or Micro-C, will be important for assessing the generality of the patterns observed here. Together, this work provides a foundational resource for studying 3D genome organization in perennial crops and a framework for future hypothesis-driven investigations of plant genome regulation.

## Materials and methods

### Hi-C library construction and data processing

Fruit and leaf tissues from *A. chinensis* cv. ‘Hongyang’ were collected, with three biological replicates per tissue. Hi-C libraries were prepared following a previously published *in situ* Hi-C protocol and sequenced on the Illumina NovaSeq 6000 platform. Raw reads were trimmed and quality-filtered using fastp (0.20.1) [59]. Clean reads were then processed using HiCExplorer (3.7.6) [60] for alignment, binning, and matrix construction. Hi-C contact matrices were generated at multiple resolutions and normalized using either the ICE or KR method, depending on downstream application requirements. Reproducibility among biological replicates was assessed using the SCC [61], and pairwise Pearson correlations were computed using the hicCorrelate function in HiCExplorer.

### CUT&tag sequencing and data processing

CUT&Tag libraries were prepared from young leaf tissues of *A. chinensis* to profile seven histone modifications: H3K4me3, H3K4me, H3K9ac, H3K27ac, H3K36me3, H3K27me3, and H3K9me3. Two biological replicates were generated for each histone mark. Approximately 1 g of fresh leaf tissue was flash-frozen in liquid nitrogen, ground into a fine powder, and resuspended in ice-cold NIB lysis buffer. The lysate was filtered and centrifuged to isolate intact nuclei, which were subsequently washed and resuspended in fresh buffer for downstream processing [62]. Purified nuclei were immobilized on concanavalin A–coated magnetic beads and incubated sequentially with primary and secondary antibodies targeting the desired histone modifications. Tagmentation was performed using pA-Tn5 transposase, followed by DNA purification, PCR amplification, and size selection using VAHTS DNA Clean Beads. Final libraries were sequenced on the Illumina NovaSeq 6000 platform in 150 bp paired-end mode. Raw reads were adapter-trimmed using fastp (v0.20.1) and aligned to the reference genome using BWA (0.7.17) [63]. Genome-wide signal coverage tracks were generated using bamCoverage (deepTools v3.5.6) [64] at 100-, 40-, and 25-kb resolutions. ATAC-seq libraries were processed using the same pipeline and visualized using pyGenomeTracks [65].

### Chromatin compartment identification and analysis

Hi-C contact matrices were KR-normalized per chromosome using HiCExplorer (v3.7.6) and aggregated into 100-kb resolution matrices. Observed/expected (O/E) contact values were calculated using hicTransform, and Pearson correlation matrices were computed between genomic bins. PCA was then performed on each chromosome, and the first principal component (PC1) was extracted to define A/B compartments [66]. PC1 vectors were manually reoriented, if necessary, to ensure that positive values corresponded to gene-rich, transcriptionally active A compartments. Compartment tracks were visualized alongside genomic features such as gene density, TE content, expression, methylation, and histone modifications using pyGenomeTracks. Due to the limited interpretability of PCA-derived compartments at finer scales, high-resolution subcompartment states were inferred using Calder [37, 67] at 100- and 40-kb resolutions. KR-normalized matrices were first batch-corrected and input into Calder to infer 2-, 4-, and 8-class hierarchical subcompartments (e.g. A1.1–B2.2) based on correlation matrix decomposition. Subcompartment assignments were used in downstream analyses to assess associations with gene expression, chromatin accessibility, DNA methylation, and histone modification profiles [25].

### TAD-like structure identification and classification

TAD-like domains were identified using hicFindTADs [60] from HiCExplorer (v3.7.6) on KR-normalized Hi-C contact matrices at 40-, 25-, and 10-kb resolutions. Domain calling was performed for each tissue (leaf and fruit) separately, using the following parameters: –minDepth 40 000, –maxDepth 400 000, –step 40 000, –correctForMultipleTesting fdr. All calls were executed with default statistical settings to control false discovery rate, and the resulting domain boundaries were output as browser extensible data (BED) files. Domain architectures were visualized using hicPlotTADs and pyGenomeTracks. To identify differentially structured TAD-like domains between leaf and fruit tissues, we applied hicDifferentialTAD to matched resolution matrices (40, 25, and 10 kb). Leaf matrices were set as the target, and fruit as the control. The corresponding leaf TAD boundary annotations were provided via the –tadDomains option. Differential analysis was performed using the –mode all, –modeReject one, and –pValue 0.05 settings.

For assessing domain coverage similarity across biological replicates and tissues, the Jaccard index $J\left(A,B\right)=\mid A\cap B\mid /\mid A\cup B\mid$ was calculated based on BED intervals using custom R scripts. Jaccard distance matrices were then used for hierarchical clustering (hclust, complete linkage) in R (4.1.2). For domain functional classification, each TAD-like region was annotated with multiple genomic and epigenomic features, including gene density, TE density, mean gene expression (TPM), DNA methylation (CG, CHG, CHH contexts from whole-genome bisulfite sequencing (WGBS)), and seven histone marks (H3K4me3, H3K4me, H3K36me3, H3K27ac, H3K9ac, H3K27me3, H3K9me3 from CUT&Tag). Features were averaged across each domain and *z*-score normalized. Euclidean distances were calculated from the resulting feature matrix, followed by unsupervised clustering using hclust. Final domain classification into Active, HDF, or IT types was performed based on multivariate epigenomic profiles, following the strategy established in pepper [25]. Classification results were visualized using the pheatmap R package.

### Chromatin loop detection and cross-tissue comparison

Merged Hi-C matrices from three biological replicates per tissue were used to annotate chromatin loops with Mustache [40] at 1-, 2-, and 5-kb resolutions. To complement loop detection, hicDetectLoops [60] from HiCExplorer was also applied to KR-normalized matrices at 5, 10, 25, and 40 kb. Loop overlap and conservation between tissues were quantified using bedtools and visualized with DeepTools [64]. To validate the reliability of loop calls and compare loop strength across tissues, we performed APA using hicAggregateContacts from HiCExplorer. APA matrices were generated by aggregating normalized contact intensities within ±100-kb windows centered at loop anchors, using 10-kb bins. Signal enrichment at loop centers was quantified by calculating the observed/expected contact ratios relative to the surrounding background. APA plots were visualized as heatmaps, and average enrichment scores were compared between fruit and leaf tissues to assess differences in loop signal intensity.

### Integration of 3D genome organization and gene expression

To explore the regulatory implications of 3D chromatin organization, all 40-kb genomic bins were categorized based on subcompartment dynamics between leaf and fruit tissues: (i) ‘down’ bins, which underwent a ≥1-level decrease in subcompartment rank (e.g. A1.1 → B2.2); (ii) ‘up’ bins, with a ≥1-level increase; and (iii) ‘stable’ bins, showing no change in classification [25]. DEGs were assigned to each category based on overlap with their TSS, and enrichment was assessed using Fisher’s exact test. Expression fold changes were calculated using DESeq2-derived values, and gene distributions were visualized using boxplots. To further evaluate the relationship between subcompartment shifts and transcription, a reciprocal analysis was performed: all expressed genes were stratified into three groups—upregulated (≥2-fold increase), downregulated (≥2-fold decrease), and stable—and their corresponding changes in subcompartment rank were quantified and compared using Wilcoxon rank-sum tests. TAD-like domains, TAD boundaries, and chromatin loops were classified as tissue-specific (present in only one tissue) or conserved (shared across tissues). DEGs were mapped to these structural categories and tested for enrichment using Fisher’s exact tests. For boundary-level comparisons, average insulation scores were used to confirm boundary identity and strength. Genes located within or adjacent to tissue-specific vs conserved boundaries were evaluated for both differential expression and expression specificity.

Tau Index [68]:

Tissue specificity was calculated using the Tau index, defined as:


$$ \tau =\frac{\boldsymbol{\sum}n-1}{\left(1- ri\right)} $$

where *ri* is the expression of a given gene in tissue *i*, normalized to its maximum expression across all *n* tissues. Tau values range from 0 (ubiquitous) to 1 (tissue-specific). Expression values were averaged across three replicates for robustness. The Tau index was compared between gene sets located within conserved vs tissue-specific TAD boundaries, as well as among genes associated with shared vs tissue-specific chromatin loops.

### RNA-seq data processing and differential expression analysis

Raw RNA-seq reads were quality trimmed using fastp (v0.20.1) with default parameters for adapter removal and low-quality base filtering. Clean reads were aligned to the reference genome using HISAT2 [69] (2.2.1) with default settings and a prebuilt splice-aware index. Gene-level read counts were generated using featureCounts [70] in exon-based mode with parameters -p -B -s 0, and gene annotations were based on the GTF file corresponding to the reference genome. DEGs were identified using DESeq2 (v1.32.0) in R, with genes filtered for a minimum average count >10. Significance thresholds were set as adjusted *P*-value (padj) <0.05 and |log2(fold change)| ≥1. Genome-wide expression coverage tracks were computed using bamCoverage (deepTools v3.5.6) with a bin size of 1 kb, normalized by FPKM, and excluding multimapping and duplicate reads.

### Whole-genome bisulfite sequencing and methylation analysis

WGBS libraries were processed using fastp for adapter trimming and quality filtering. Reads were aligned to the bisulfite-converted version of the reference genome using Bismark (0.23.1) [71] with —bowtie2 and —non_directional options enabled. Duplicates were removed using deduplicate_bismark. Cytosine methylation calls were extracted using bismark_methylation_extractor, and genome-wide methylation statistics were obtained. Context-specific methylation levels (CG, CHG, CHH) were calculated using methylKit (0.99.2) [72]. Only cytosines with ≥5× coverage and <99th percentile coverage were retained. Methylation levels were summarized in 100-bp or 1-kb windows for visualization and integration with 3D chromatin features.

### Identification and mapping of fruit-specific genes to 3D chromatin features

We constructed a transcriptome matrix using RNA-seq data from multiple tissues and fruit developmental stages of *A. chinensis*, and identified broadly fruit-specific genes. The selection criteria were: (i) TPM >1 in all fruit samples; (ii) TPM <0.5 in nonfruit tissues (e.g. root, leaf, flower); (iii) tissue specificity index Tau ≥0.6; and (iv) fruit-derived transcripts accounted for >80% of total expression. A total of 68 fruit-specific genes were retained for downstream analysis.

The genomic coordinates of these genes were projected onto Hi-C maps from both fruit and leaf tissues to examine their spatial chromatin context. TAD-like domains, A/B compartment annotations, and chromatin loops were integrated to comprehensively assess their 3D structural positioning. For TAD analysis, HiCExplorer-derived annotations at 40-kb resolution were used to determine whether each gene was located within a TAD, and the length of the associated domain was compared across tissues. TAD boundary insulation strength was evaluated using hicCalculateInsulationScore, and the average insulation score was calculated over a ±400-kb window (21 bins) centered at each gene’s domain boundary.

For compartment analysis, 40-kb bins containing the fruit-specific genes were assigned to A or B subcompartments based on PC1 direction and Calder predictions. Subcompartment distributions were compared between fruit and leaf tissues, and potential compartment switching events were recorded.

Chromatin loops were derived from the consensus of Mustache and hicDetectLoops across multiple resolutions. Loop anchors were defined as ±5-kb windows around loop midpoints. Gene-loop overlaps were identified using bedtools intersect, and loops were categorized as fruit-specific, leaf-specific, or shared. The relationship between loop association and gene expression was examined using contingency tables and visualized using R.

## Supplementary Material

Web_Material_uhag076

## Data Availability

All sequencing data supporting this study have been deposited in public repositories. The Hi-C and CUT&Tag (seven histone modifications) datasets generated in this study are available from the China National GeneBank Database (CNGBdb; https://db.cngb.org/) under accession number CNP0007920. Chromatin accessibility (ATAC-seq) data and fruit RNA-seq data across multiple developmental stages are available from the Genome Sequence Archive (GSA; https://ngdc.cncb.ac.cn/gsa/) under accession number PRJCA024619 and PRJCA003268, respectively [73, 74]. WGBS data, along with RNA-seq data from root, stem, leaf, and flower tissues are available from NCBI BioProject under accession numbers PRJNA1039995, PRJNA328414 and PRJNA389468, respectively [75–77].
