# Supplementary material for: A high-resolution 3D genome map of kiwifruit provides insights into chromatin architecture and transcriptional activity
Source: Hortic Res. 2026 Jun 2;13(6):uhag076. doi: 10.1093/hr/uhag076 (PMC13253338; doi:10.1093/hr/uhag076)
Supplement: Web_Material_uhag076 [file web_material_uhag076.zip › SupplementaryFigures.R1.pdf]

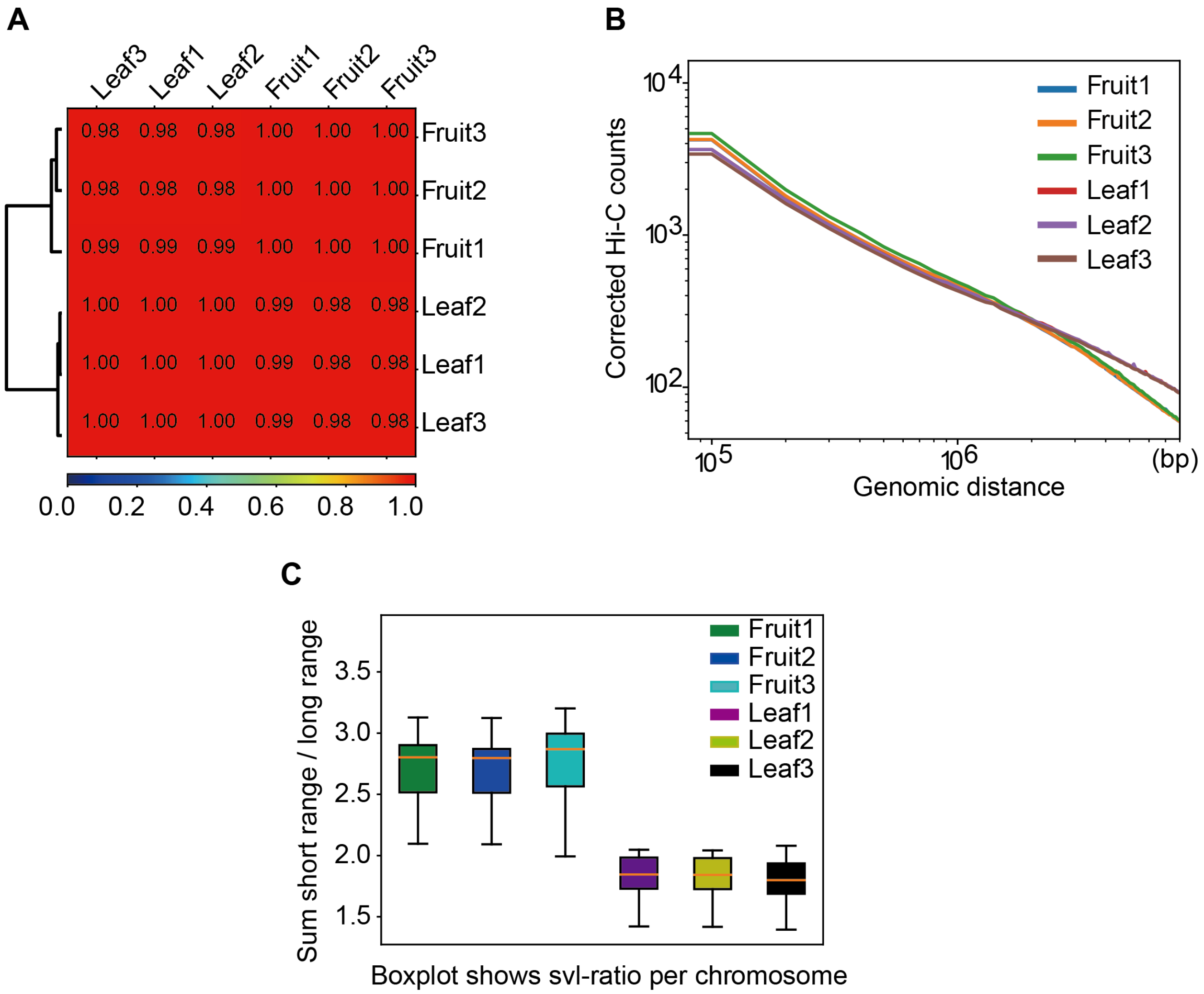

Supplementary Figure 1 Reproductivity and interaction decay across Hi-C libraries. (A) Pearson correlation heatmap of all Hi-C replicates, confirming high reproducibility (*r* > 0.92). **(B)** Contact decay curves plotted for each biological replicate, showing consistent interaction profiles across fruit and leaf samples. **(C)** Boxplots of short-to-long range interaction ratios per chromosome across six replicates. Fruit samples show significantly higher levels of short-range interaction enrichment than leaf samples.

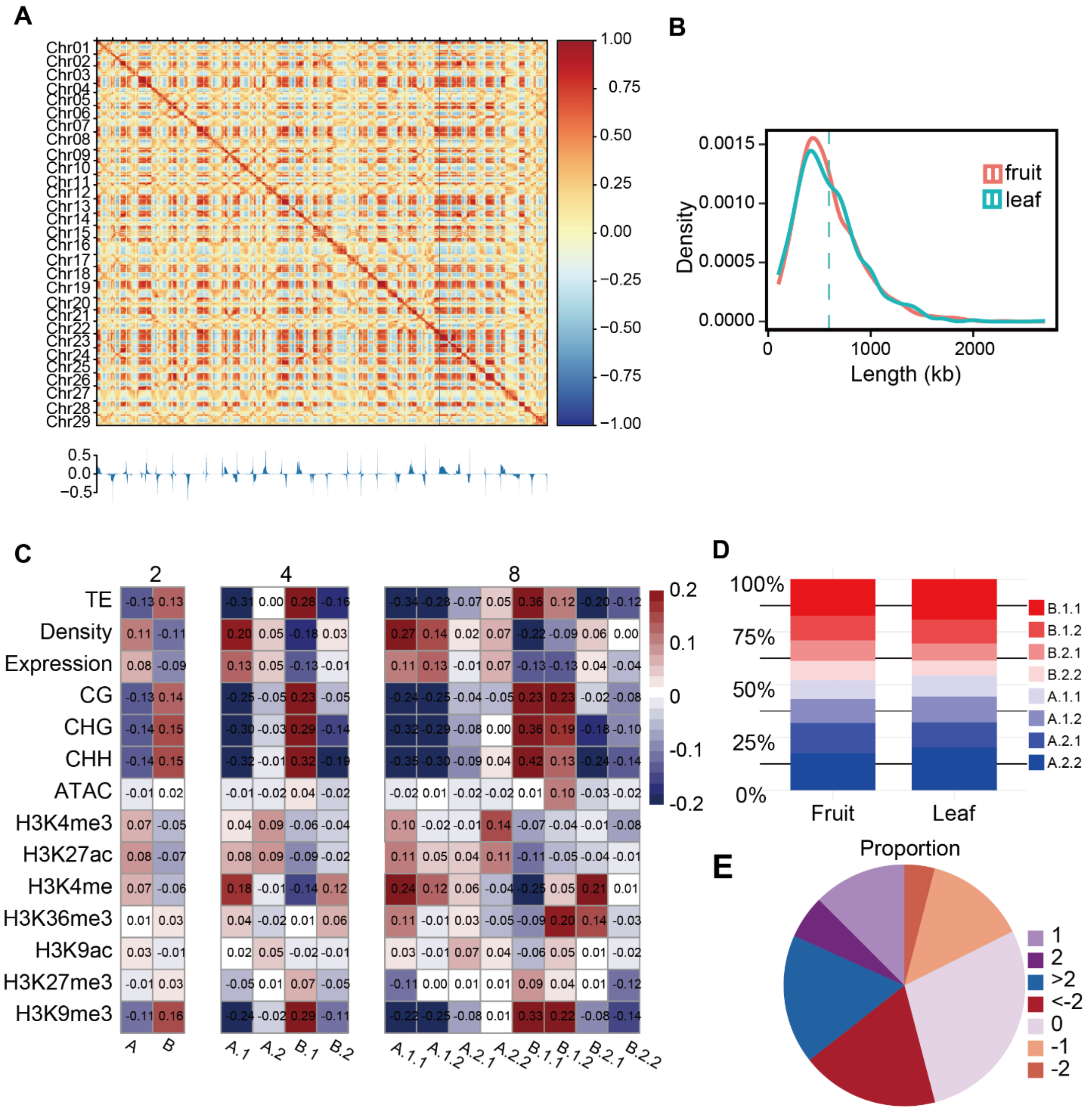

Supplementary Figure 2 Resolution-specific properties and cross-tissue conservation of chromatin subcompartments. (A) Genome-wide A/B compartment classification at 250-kb resolution in fruit tissue. PC1 values were used to define A (positive) and B (negative) compartments. **(B)** Length distribution of subcompartments inferred by Calder at 100-kb resolution, showing broader average domain size compared to higher resolution results. **(C)** Pearson correlation heatmap showing the associations between eight subcompartment classes (A1.1 to B2.2) and various genomic and epigenomic features (e.g., gene expression, TE density, DNA methylation, histone modifications) in fruit and leaf tissues, based on 100-kb resolution data.

(D) Cross-tissue comparison of subcompartment annotations between fruit and leaf at 100-kb resolution. **(E)** Transition dynamics of subcompartments between fruit and leaf at 100-kb resolution. Bars indicate bins with unchanged subcompartment assignments (“0”), shifts spanning one (“±1”) or two (“±2”) hierarchical levels, or transitions involving more than two levels (“>±2”). Positive (“+”) and negative (“−”) values denote shifts toward higher (e.g., A.1.1) or lower (e.g., B.2.2) subcompartment ranks, respectively.

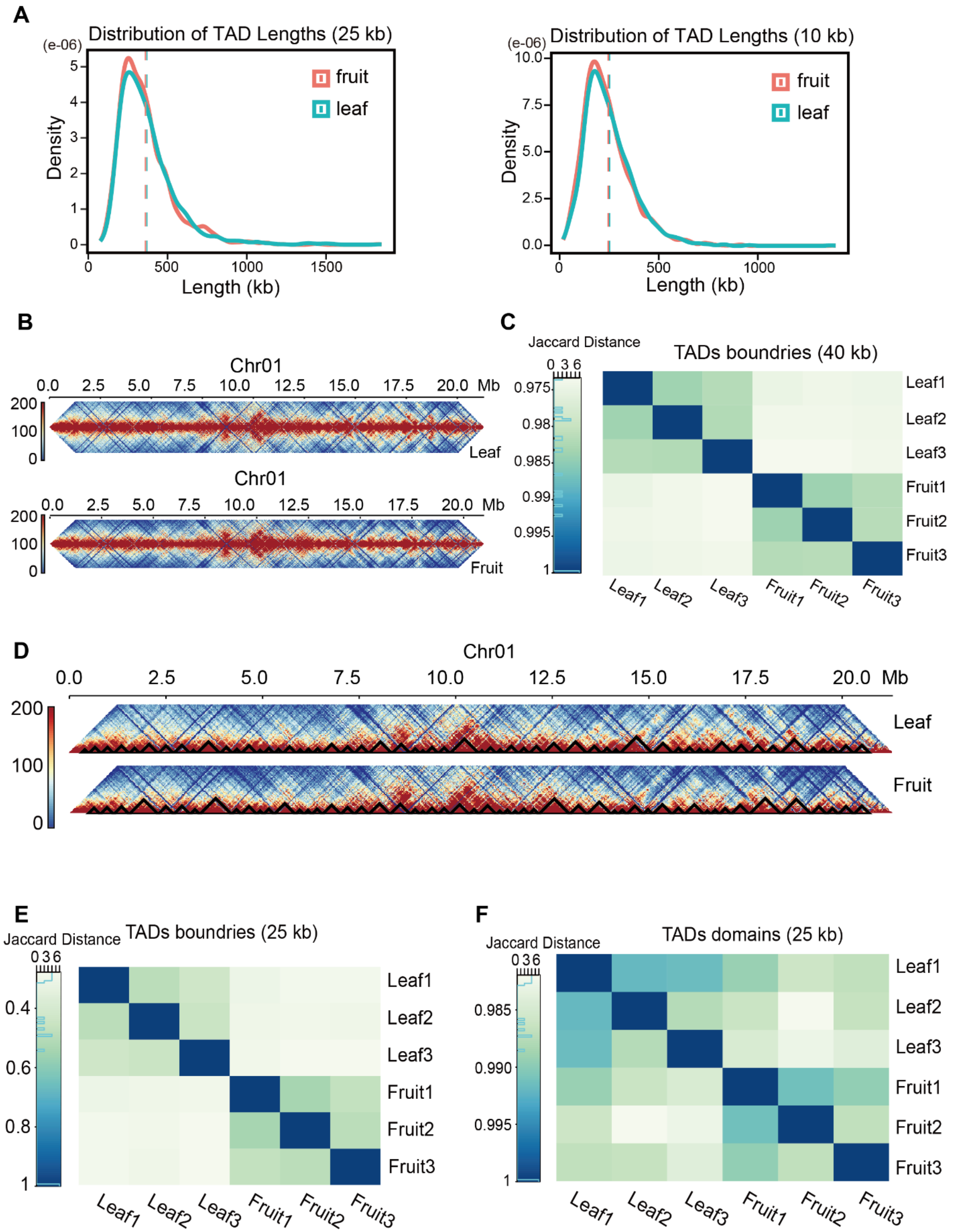

Supplementary Figure 3 Evaluation of resolution and tissue effects on TAD-like domain identification. **(A)** Domain length distributions in leaf tissue at 10-kb and 25-kb resolutions. **(B)**

Hi-C contact maps (Chr01) at 40-kb resolution showing annotated TAD-like domains in leaf and fruit tissues. **(C)** Jaccard similarity comparison of domain boundaries between leaf and fruit tissues at 40-kb resolution. **(D)** Genome browser view of representative regions (25-kb resolution) highlighting conserved and tissue-specific domain boundaries between leaf and fruit tissues. **(E)** Jaccard distance matrix showing pairwise overlap between domain boundaries across all samples. **(F)** Jaccard distance matrix assessing overall consistency of domain calls between biological replicates and tissues.

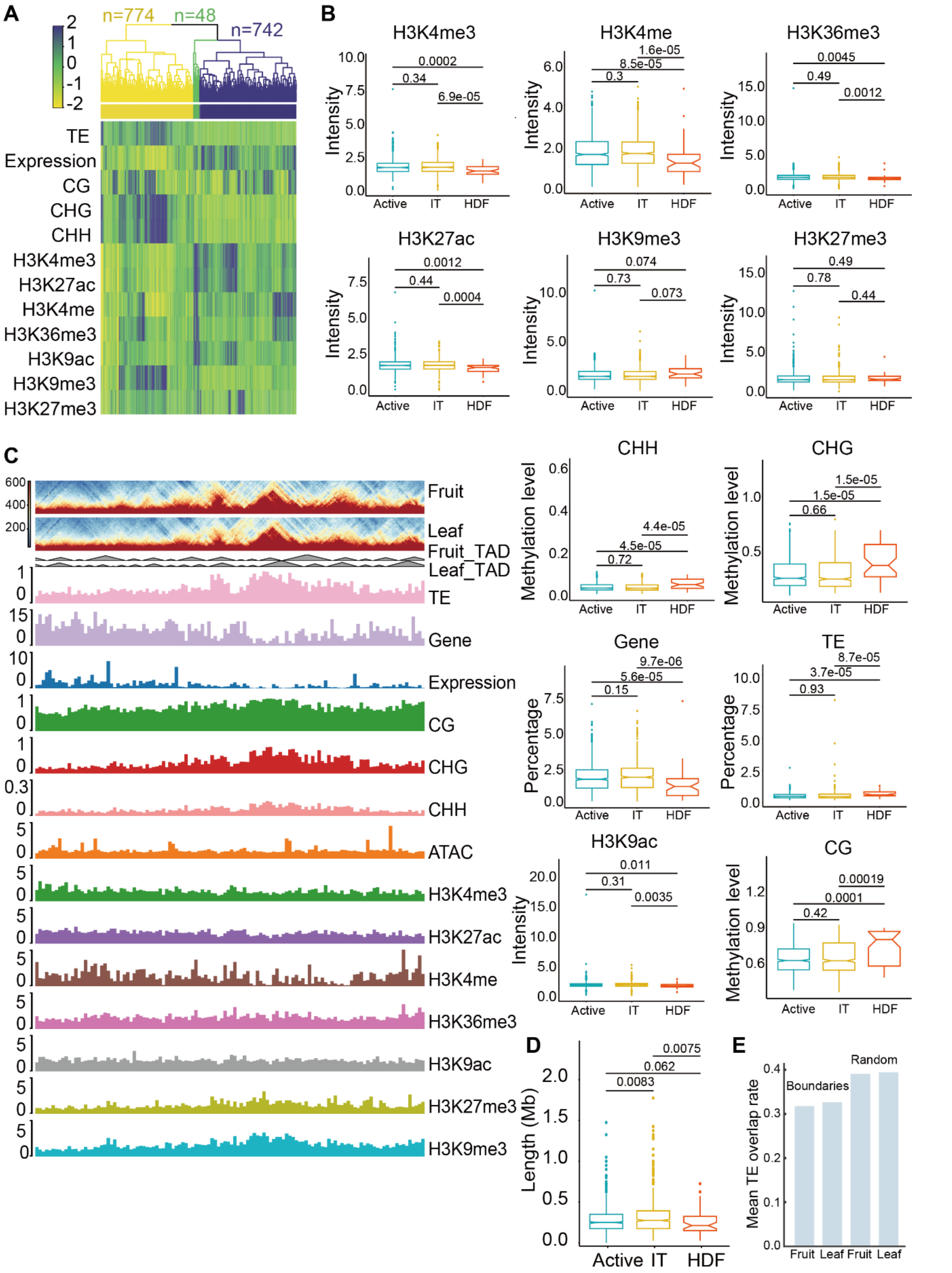

Supplementary Figure 4 Classification of TAD-like domains in kiwifruit leaf tissue at 25-kb resolution. (A) At 25-kb resolution, TAD-like domains were classified into three categories:

Active, Intermediate (IT), and Heterochromatin-Driven Folding (HDF). **(B)** Multi-omics heatmap showing differential enrichment of genomic and epigenomic features across the three domain types at 25-kb resolution. **(C)** Genome browser view (40-kb resolution) illustrating concordance between domain boundaries and genomic/epigenomic features. **(D)** Length distribution of Active, IT, and HDF domains at 25-kb resolution. **(E)** Overlap between TEs and domain boundaries (±40 kb).

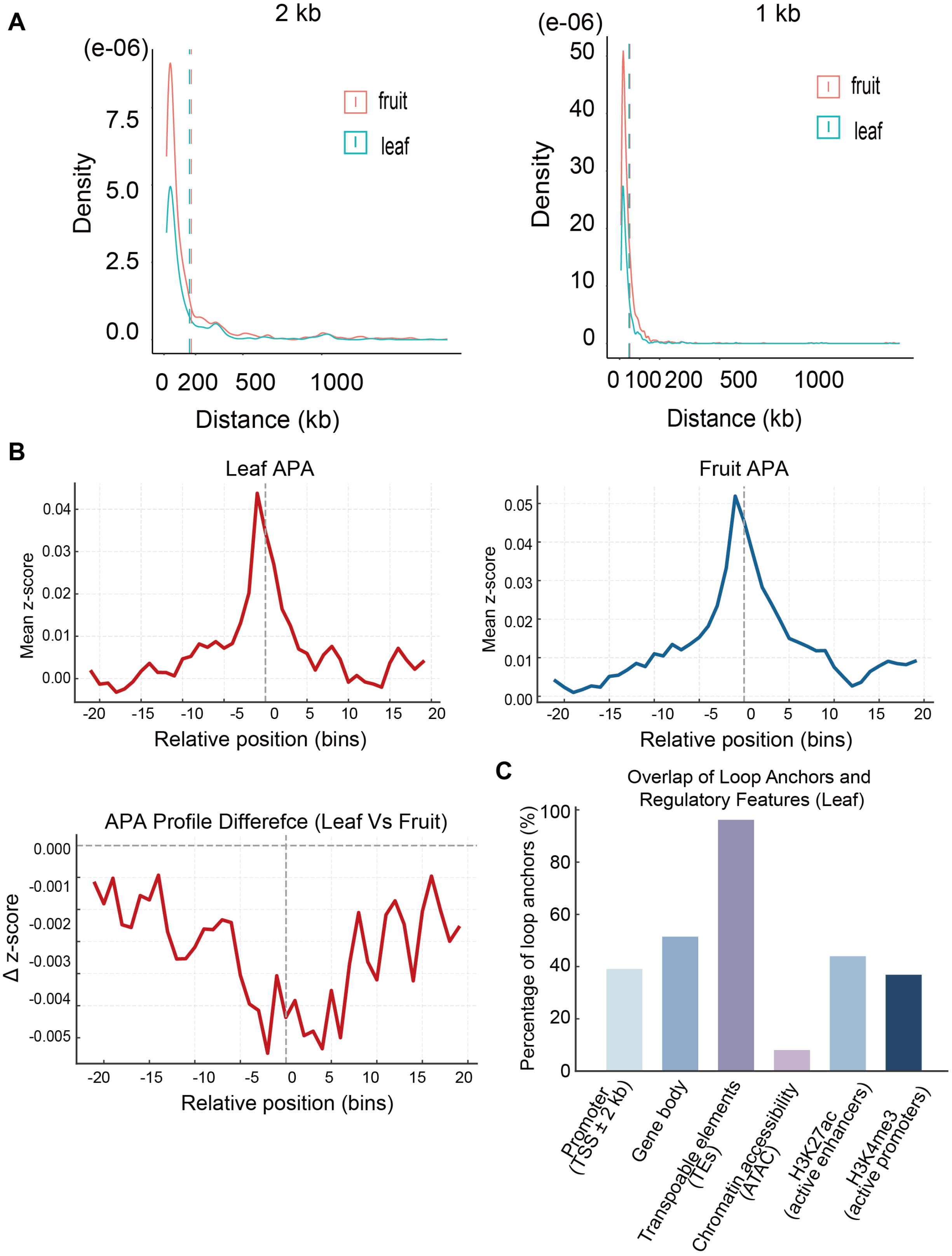

Supplementary Figure 5 Chromatin loop cross-validation and analysis. (A) Over 80% of loops identified in both tissues span less than 500 kb. (**B**) APA analysis of loop anchors in fruit and leaf

showing interaction enrichment and reproducibility. **(C)** Functional annotation of chromatin loop anchors in leaf tissue. Bar plot showing the percentage of loop anchors overlapping promoters (TSS±2kb), gene bodies, TEs, ATAC peaks, H3K27ac peaks, and H3K4me3 peaks. Given that these features are not mutually exclusive, individual loop anchors may overlap multiple categories.

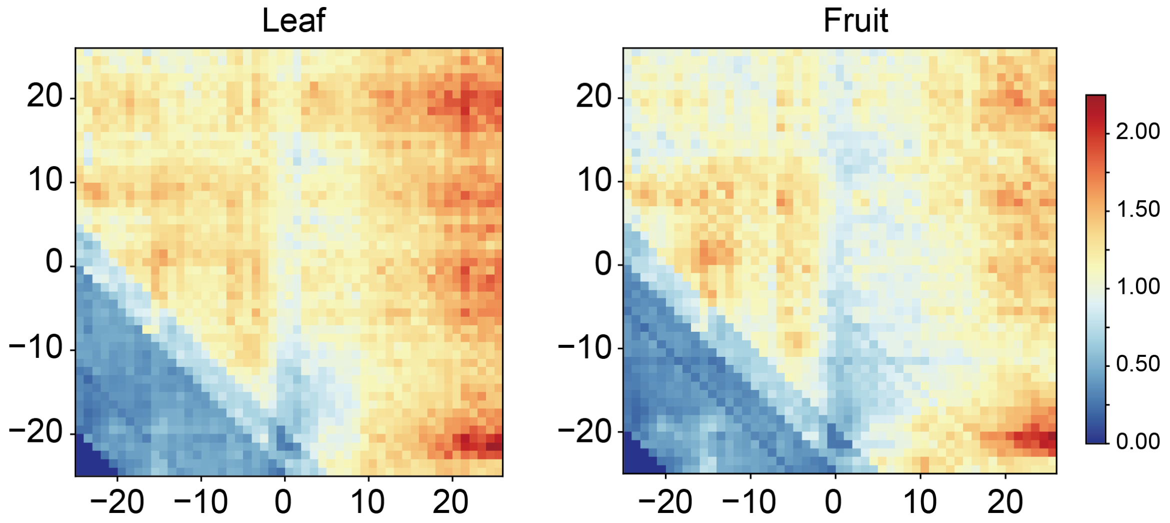

Supplementary Figure 6 APA analysis of pericentromeric regions. APA was performed using KR-normalized Hi-C matrices at 40-kb resolution with a ±50-bin window centered on pericentromeric (periCEN) intervals. The resulting observed/expected heatmap shows smooth short-range interaction decay without focal contact enrichment, indicating that periCEN heterochromatin adopts a compact yet structurally stable conformation in kiwifruit.
