# Supplementary material for: A high-resolution 3D genome map of kiwifruit provides insights into chromatin architecture and transcriptional activity
Source: Hortic Res. 2026 Jun 2;13(6):uhag076. doi: 10.1093/hr/uhag076 (PMC13253338; doi:10.1093/hr/uhag076)
Supplement: Web_Material_uhag076 [file web_material_uhag076.zip › SupplementaryTables.R1.pdf]

Supplementary Table 1. Statistics of Hi-C libraries

	**Leaf (Rep1)**	**Leaf (Rep2)**	**Leaf (Rep3)**	**Fruit (Rep1)**	**Fruit (Rep2)**	**Fruit (Rep3)**
**Raw read pairs**	1,203,575,679	1,223,236,567	826,740,396	1,344,645,866	1,023,839,092	890,662,178
**Unique mapping**	881,458,176 (73.24%)	898,091,631 (73.42%)	600,849,928 (72.68%)	971,793,854 (72.27%)	727,106,763 (71.02%)	619,534,270 (69.56%)
**Hi-C contacts**	588,508,854 **(48.89%)**	603,374,779 **(49.33%)**	401,701,461 **(48.59%)**	626,435,691 **(46.76%)**	478,346,884 **(46.72%)**	437,513,004 **(49.13%)**
**Inter-chromosomal contacts**	308,614,043 (52.44%)	316,986,684 (52.53%)	215,603,249 (53.67%)	322,808,933 (51.53%)	244,266,577 (51.06%)	214,091,444 (48.93%)
**Intra-chromosomal contacts**	572,844,133 (47.56%)	581,104,947 (47.47%)	385,246,679 (46.33%)	648,984,921 (48.47%)	482,840,186 (48.94%)	405,442,826 (51.07%)
**Intra-chromosomal short range (< 20kb)**	51,024,547 (8.67%)	51,797,715 (8.58%)	35,246,888 (8.77%)	79,149,079 (12.59%)	59,851,442 (12.51%)	54,114,721 (12.37%)
**Intra-chromosomal long range (≥ 20kb)**	228,824,089 (38.89%)	234,653,400 (38.89%)	150,839,942 (37.55%)	226,743,047 (36.07%)	174,254,393 (36.43%)	169,333,539 (38.70%)

Supplementary Table 2. Resolution of Hi-C contact maps across six samples. Map resolution is defined as the smallest locus size at which 80% of loci have at least 1,000 contacts. Based on this criterion, the map resolution across the six samples ranged from 2 kb to 10 kb. At a 1-kb bin size, all samples exhibited slightly lower coverage than the 2-kb threshold.

Samples/Tissues	1-kb	2-kb	5-kb	10-kb
Fruit 1	422313/608340	273453/304177	116736/121679	59375/60847
	**69.42%**	**89.90%**	**95.94%**	**97.58%**
Fruit 2	413951/608340	272783/304177	116751/121679	59371/60847
	**68.05%**	**89.68%**	**95.95%**	**97.57%**
Fruit 3	407768/608340	270971/304177	116750/121679	59359/60847
	**67.03%**	**89.08%**	**95.95%**	**97.55%**
Leaf 1	431329/608340	274711/304177	116787/121679	59383/60847
	**70.90%**	**90.31%**	**95.98%**	**97.59%**
Leaf 2	434307/608340	275001/304177	116801/121679	59387/60847
	**71.39%**	**90.40%**	**95.99%**	**97.60%**
Leaf 3	422143/608340	274190/304177	116746/121679	59368/60847
	**69.39%**	**90.14%**	**95.95%**	**97.57%**

Supplementary Table 3. Loops identified at different resolution in leaf and fruit tissues

Tissue	5-kb	10-kb	25-kb	40-kb	Merged
Leaf	1013	498	100	44	1655
Fruit	1378	681	144	74	2277

Supplementary Table 4. Overlap of DEGs with different chromatin loops.

Loop type	Total loops	Loops overlapping with DEGs	%DEG Overlapped
Shared	1480	634	42.80%
Tissue-specific (Leaf + Fruit)	3907	1628	41.70%
Total	5387	2262	42.00%
